# Characterization and Modeling of Reversible Antibody Self-Association Provide Insights into Behavior, Prediction, and Correction

**DOI:** 10.3390/antib10010008

**Published:** 2021-02-15

**Authors:** Carl Mieczkowski, Alan Cheng, Thierry Fischmann, Mark Hsieh, Jeanne Baker, Makiko Uchida, Gopalan Raghunathan, Corey Strickland, Laurence Fayadat-Dilman

**Affiliations:** 1 Discovery Biologics, Protein Sciences, Merck & Co., Inc., South San Francisco, CA 94080, USA; carl.mieczkowski@merck.com (C.M.); mark_hsieh@merck.com (M.H.); jeanne.baker@merck.com (J.B.); makiko.uchida@merck.com (M.U.); Raghu108@gmail.com (G.R.); laurence.fayadat-dilman@merck.com (L.F.-D.); 2 Discovery Chemistry, Modeling and Informatics, Merck & Co., Inc., South San Francisco, CA 94080, USA; 3 Department of Chemistry, Modeling and Informatics, Merck & Co., Inc., Kenilworth, NJ 07033, USA; thierry.fischmann@merck.com (T.F.); corey.strickland@merck.com (C.S.)

**Keywords:** antibody, protein, self-association, self-interaction, developability, in silico prediction, computational modeling, viscosity, dynamic light scattering

## Abstract

Reversible antibody self-association, while having major developability and therapeutic implications, is not fully understood or readily predictable and correctable. For a strongly self-associating humanized mAb variant, resulting in unacceptable viscosity, the monovalent affinity of self-interaction was measured in the low μM range, typical of many specific and biologically relevant protein–protein interactions. A face-to-face interaction model extending across both the heavy-chain (HC) and light-chain (LC) Complementary Determining Regions (CDRs) was apparent from biochemical and mutagenesis approaches as well as computational modeling. Light scattering experiments involving individual mAb, Fc, Fab, and Fab’2 domains revealed that Fabs self-interact to form dimers, while bivalent mAb/Fab’2 forms lead to significant oligomerization. Site-directed mutagenesis of aromatic residues identified by homology model patch analysis and self-docking dramatically affected self-association, demonstrating the utility of these predictive approaches, while revealing a highly specific and tunable nature of self-binding modulated by single point mutations. Mutagenesis at these same key HC/LC CDR positions that affect self-interaction also typically abolished target binding with notable exceptions, clearly demonstrating the difficulties yet possibility of correcting self-association through engineering. Clear correlations were also observed between different methods used to assess self-interaction, such as Dynamic Light Scattering (DLS) and Affinity-Capture Self-Interaction Nanoparticle Spectroscopy (AC-SINS). Our findings advance our understanding of therapeutic protein and antibody self-association and offer insights into its prediction, evaluation and corrective mitigation to aid therapeutic development.

## 1. Introduction

Monoclonal antibodies and biologics in general have enjoyed increasing success and utility as therapeutic agents addressing a variety of biological targets of interest. As of late 2019, 79 commercial monoclonal antibody or antibody-based therapeutics have been approved [1], with several hundred currently being evaluated in clinical development [2]. Central to a therapeutic antibody’s selection and success is its developability profile, which is a key driver in pre-clinical and clinical lead nomination [3,4]. Previously, developability flags in therapeutic antibodies have been correlated to overall clinical success, clearly indicating that developability attributes may impact clinical development beyond drug product purity, stability and manufacturability [5]. The developability properties of therapeutic antibodies range from expression and purification amenability to its physicochemical stability and behavior, both in the drug product form and in vivo [6,7,8]. Other major developability properties, such as self-association, can directly impact manufacturability and formulation success [3,9], and even strongly correlate to non-specific binding and animal Pharmacokinetics and clearance, therefore, affecting its overall efficacy [10,11,12,13].

Therapeutic antibody self-association has been well studied from a rheological standpoint and is known to directly impact solution viscosity, injectability, and manufacturability [14,15]. Therapeutic antibody formulations as low as 13 mg/mL have been reported to appreciably self-associate, significantly increasing solution viscosity and decreasing solubility, precluding further development even at typical dose concentrations and formulation conditions [16]. Moreover, strong antibody self-interaction tends to manifest in high viscosities at higher formulation concentrations, such as 100 to 200 mg/mL (or approximately 0.7–1.4 mM for a typical monoclonal antibody) and beyond [3]. Increasing solution viscosity is due to a concentration-dependent oligomerization effect of self-interacting molecules, particularly antibodies, whereby effectively large polymeric structures give rise to dramatic changes in solution rheology [17,18]. A resulting increase in viscosity may be exponential, making process filtration and pumping operations difficult and infeasible, and in the drug product form, handling, injectability, and potentially even stability may be negatively impacted [3,19,20]. This behavior is a major negative developability attribute that is difficult to predict from sequence or structure and correct through molecular engineering and can halt further development and the selection of even the most promising large-molecule candidates [21].

High-concentration rheological behavior has significant importance in the selection of lead therapeutic candidates [22]. In general, molecular properties such as pI, net charge, and hydrophobicity can affect the rheology of antibody solutions [23]. Particularly at higher drug concentrations, it was shown that hydrophobic and charged surface patches result in increased self-interaction and solution viscosity above 100 mg/mL and approaching 200 mg/mL [24,25]. Such self-interactions can even lead to additional undesirable outcomes, such as opalescence, phase separation and gelling [26,27]. Antibody self-interactions have been reported to occur between antibody variable domains [21,28] as well as variable–constant interactions [16]. To possibly predict or correlate molecular properties to rheological outcomes, in silico computational approaches have been employed to ascertain a molecule’s propensity to self-interact [29]. Surface behavior characteristics, such as zeta potential and net charge derived from modeling, have also been correlated to viscosity, [18,30,31] other self-interaction parameters, including AC-SINS and kD [32], and lead molecule selection and success [33,34]. In addition to the inherent or predicted properties of a molecule, temperature [35,36] and formulation conditions [9,28] can have a dramatic effect on solution rheology. Because of this, formulation approaches have been successful in mitigating self-association, such as modulating pH and ionic strength [37,38,39], or the addition of excipients, such as Arginine [40,41]. However, success is still dependent on the nature of the self-interacting therapeutic molecule and additional challenges and limitations are presented both from a process and formulation perspective.

In addition to directly measuring viscosity, a number of analytical methods and characteristics are informative of self-association, where typically size is directly measured or characterized. Notably, light scattering approaches, such as Dynamic Light Scattering (DLS), to determine the diffusion interaction parameter (kD) are informative and have been correlated to viscosity at higher concentrations [42]. Other measured or calculated characteristics, such as the second virial coefficient (A_2_ or B_22_), isoelectric point (pI) and zeta potential, have also been correlated to self-association phenomena [42,43,44]. Other techniques that evaluate self-interaction propensity or the propensity to interact with a column matrix involve Affinity-Capture Self-interaction Nanoparticle Spectroscopy (AC-SINS) [45,46,47], Analytical Ultracentrifugation (AUC) [48,49], Cross-Interaction Chromatography (CIC) and Standup Monolayer Adsorption Chromatography (SMAC) [43], many of which have been previously correlated to other self-interaction parameters, such as kD, B_22_ and viscosity [39,50,51].

While self-association is impacted and defined by molecular surface properties, formulation effects, process considerations, and characterization approaches, the fundamental nature of an antibody’s reversible self-interaction has yet to be fully gleaned, such as its typical orientation and strength. Additionally, while self-association can be routinely characterized and has been correlated to surface attributes elucidated both from crystal structures and computational models [16,21,29,52], predicting self-association from sequence or using in silico techniques to address and mitigate it is not routine or fully understood. Therefore, there is significant interest in understanding how to better predict and correct self-association at a sequence level to advance developability efforts. Previously, self-association of a recombinant monoclonal antibody was dramatically impacted and modulated by conservative mutagenesis of a single heavy-chain complementary determining region-3 (HC-CDR3) residue at position 104 [6], necessitating mitigation to find variants with lower, more acceptable self-association propensity. Developability attributes were evaluated for these variants, including DLS, AC-SINS, and viscosity, and correlations between these methods were apparent. However, this case study also offered a unique opportunity to further probe and to examine the intrinsic nature of self-interaction and how to better predict and correct it. Herein using similar variants, we verified viscosity profiles in a representative formulation buffer using the W104 and F104 mAb variants, validating the strongly self-associating F104 variant as a major developability risk to formulation efforts. We also mapped this strong self-association by evaluating the interaction of individual antibody domains using DLS, revealing this self-interaction was independent of the Fc domains and involved a blocking or likely face-to-face interaction between opposing Fab domains involving the CDR apparatus. We also determined the binding affinity of this strong self-interaction using both BIAcore Surface Plasmon Resonance (SPR) and Isothermal Titration Calorimetry (ITC), revealing, for the first time, the magnitude and binding affinity of representative, strong antibody self-interaction that arises in unacceptably high viscosity. We further evaluated in silico computational approaches, such as homology modeling and docking for their ability to predict and inform corrective engineering. Identified by homology modeling and docking were multiple key interacting residues in the HC and LC CDRs. These residues were subsequently and individually mutated, and all were found to dramatically affect self-interaction by DLS and AC-SINS. Given the highly specific and tunable nature of this self-interaction that structurally overlaps with its functionality, corrective engineering, while maintaining critical developability attributes such as target antigen binding, is challenging although demonstrated herein to be feasible. This study sheds much needed light on the nature of antibody self-association and how to potentially predict, correct and mitigate it.

## 2. Materials and Methods

### 2.1. Protein Expression and Purification

All recombinant antibodies (mAbs) and antibody binding fragments (Fabs) used are of the IgG1 isotype and were constructed by gene synthesis and expressed and purified as previously described [6]. Briefly, multi-liter (large) scale transient transfections were performed in 1 L shake flasks using the ExpiCHO Expression System (Thermo Fisher Scientific, Waltham, MA, USA) according to the manufacturer’s protocol for all protein production. Antibodies were then affinity purified by Protein A MabSelect SuRe LX resin (GE Healthcare) in batch binding mode. Eluted antibody was then buffer exchanged into 20 mM Sodium Acetate pH 5.5 or 1X PBS pH 7.4 (Thermo Scientific). All purified recombinant antibodies were buffer exchanged overnight using 10 K MWCO Slide-A-Lyzer dialysis cassettes. Samples were concentrated using a Vivaspin™ ultrafiltration spin column with 10 K MWCO membrane (Sigma, Saint Louis, MO, USA). Concentration was determined by UV absorbance at 280 nm on a Nanodrop 2000 1-position Spectrophotometer (Thermo Scientific, Waltham, MA, USA).

For the preparation of Fab’2 constructs, the relevant mAb was digested by immobilized pepsin using the Pierce F(ab’)2 Preparation Kit (Thermo Scientific, Waltham, MA, USA) according to the manufacturer protocol. Digested material was ProA affinity purified and the digested Fab’2 was recovered in the flow-through and buffer-exchanged, concentrated, and measured for concentration as performed for Fabs/mAbs.

All antibody variant identities were confirmed by intact LC-MS, as previously described [6].

### 2.2. Dynamic Light Scattering (DLS)

A DynaPro PlateReader II (Wyatt Technology, Santa Barbara, CA, USA) was used for all DLS experiments. First, 65 μL of recombinant antibody solution in 1X PBS pH 7.4 was added in duplicate to a 384-microwell glass bottom plate (Greiner bio-one, Austria). Then, 10 acquisitions were taken for a duration of 5 s at 25 °C using an auto-attenuated laser wavelength of 825 nm. Dynamics software version 7.8 (Wyatt Technology, Santa Barbara, CA, USA) was used for data analysis. All values, including hydrodynamic radius (R_h_), diffusional coefficients (D), and diffusion interaction parameters (kD) are reported as the average of duplicate well collections. kD’s in units of mL/g were obtained over the concentration range of 1–10 mg/mL at 1, 2, 4, 6, 8, and 10 mg/mL concentrations. kD was then calculated by the Dynamics software from plotting measured diffusional coefficients (D_m_) versus sample concentration (C) and using the equation D_m_ = D_o_(1 + kD·C) [53]. Negative kD’s indicate self-interaction and increases in the magnitude indicate increased self-interaction. The M_w_-R (or estimated molecular weight from radius) is calculated in Dynamics from measured diffusional coefficients using a standard spherical model.

### 2.3. Affinity-Capture Self-Interaction Nanoparticle Spectroscopy (AC-SINS)

Gold nanoparticles (Ted Pella) were exchanged into water and an 80/20 (*v*/*v*) capture antibody/non-capture antibody mixture (Jackson Immuno Research Labs, West Grove, PA, USA) was exchange into 20 mM Sodium Acetate pH 4.3. Then, 1 mL of gold nanoparticles were incubated overnight with 100 μL of antibody mixture. Gold nanoparticles were then pelleted by centrifugation and supernatant was decanted to achieve a final volume of 50 μL, followed by gentle mixing. Then, 5 μL of this concentrated nanoparticle suspension was added to 45 μL PBS solution containing 0.05 mg/mL of antibody of interest in a 384-well clear plate (Fisher Scientific, Waltham, MA, USA) and incubated at room temperature for 2 h in the dark. The plate was then quickly spun down at 3000 rpm and scanned from 450 to 650 nm using an EPOCH/2 Microplate reader from BioTek (Winooski, VT, USA). Values reported are averages of duplicate wells and are sample red shift wavelengths at maximum absorbance subtracting the blank reference (PBS only). Greater red shifts indicate increased self-interaction.

### 2.4. Viscosity Measurements

Viscosity was performed as previously described [6]. Briefly, dynamic viscosities were measured using a VROC initium (Rheosense, San Ramon, CA, USA) and processed using built-in software. Each reported viscosity value in Centipoise (cP) was the average of 10 measurements performed at 25 °C. For samples at 100 mg/mL, duplicate sample injections were performed.

### 2.5. Modeling

All modeling, including homology modeling, surface patch analysis, and protein–protein docking, was performed using MOE 2019.0102 (Chemical Computing Group, Montreal, QC, Canada), as described below. All calculations were performed using the Amber10:EHT force-field.

Homology models were produced for both F104 and W104 Fabs. The framework regions were modeled using the human antibody structure with highest sequence identity (PDB ID: 3sqo). Light chain CDR1, CDR2, and CDR3 were modeled using antibody fragment structures with PDB ID’s of 5ken, 4 × 80, and 1 × 4, with sequence identities of 82%, 100%, and 78%, respectively. Heavy chain CDR1 and CDR2 were modeled using a structure with PDB ID of 5gs2, with sequence identities of 80% and 71%, respectively. Modeling of HC-CDR3 is known to be more challenging due to higher sequence variation and flexibility. Here, we used the three structural templates with highest sequence identity, 60% (PDB IDs of 1jgu, 1dbj, and 3ixt). The three homology models produced by MOE had similar loop conformations and the positioning of the heavy chain 104 residue, and so the model based on the 3ixt template, which has a Phe at the 104 position, was selected for the F104 Fab. For the W104 Fab, we used an identical approach but selected the 1dbj template for HC-CDR3 because it has a Trp at the heavy chain 104 position.

Surface patch analysis was performed in MOE on homology models of the F104 and W104 Fabs, using the default potential threshold value of 0.09 kcal/mol.

Protein–protein docking, as implemented in MOE, was used to model the self-interaction of the F104 Fab. The approach uses a coarse-grained representation for initial docking, followed by the refinement of docking poses using an all-atom representation. Docking was limited to the CDR regions, included side-chain flexibility, and used a docking potential that includes an extra term favoring the burial of hydrophobic patches identified by the surface patch analysis. This resulted in 94 docked models, which were then clustered by binding epitopes. The largest cluster identified by this method included 14 models and involved all four VL CDR residues and at least three of the five VH CDR residues of the largest hydrophobic patch. The 14 docked poses were visually inspected and found to cluster into four binding modes.

### 2.6. Isothermal Titration Calorimetry (ITC)

Isothermal titration calorimetry (ITC) was conducted using a MicroCal PEAQ-ITC Automated (Malvern Inc., Westborough, MA, USA) to determine the dissociation constant of antibody dimers. Fabs were prepared in PBS pH 7.4. The W104 Fab (371 μM) or F104 Fab (300 μM) in the ITC syringe was titrated into the matching buffer in the ITC cell at 25 °C. Reference power was set to 10 μcal/sec with initial delay of 60 s and stirring speed of 750 rpm. Injection volume was 0.4 μL for the first injection and 4 μL for subsequent injections and 13 total injections were made. Injection duration was 0.8 s for the first injection and 6 s for subsequent injections with 150 s spacing. Baseline was adjusted using buffer–buffer titration. Data analysis was done using MicroCal PEAQ-ITC Analysis Software using the dissociation model.

### 2.7. Surface Plasmon Resonance (BIAcore)

Binding affinity of Fab against captured mAb was determined by surface plasmon resonance (SPR) on a BIAcore T200 (Cytiva). The running buffer, 10 mM HEPES, 150 mM NaCl, 0.05% *v/v* Surfactant P20, 3 mM EDTA, pH 7.4 (HBS-EP+, Cytiva, Marlborough, MA, USA) was used for immobilization and reagent dilutions. All binding kinetics were measured at 25 °C.

For each injection cycle, mAbs were first captured in flow cells 2, 3 and 4 with an anti-human Fc antibody (Human Antibody Capture Kit, Cytiva, Marlborough, MA, USA ) immobilized to the sensor chip (Series S CM5, Cytiva, Marlborough, MA, USA ). Flow cell 1 with no captured mAb was used as a reference. Serial dilutions of the Fab, ranging in concentration from 1 to 32 μM, and buffer blanks were injected in multiple cycles over the captured mAbs and reference surfaces for a 60 s association followed by a 180 s dissociation. The surfaces were regenerated with a 30 s injection of 3 M MgCl_2_ after each cycle.

Double referenced titration data were globally fit to a 1:1 Langmuir binding model to determine the association rate constant, ka (1/M·s), and the dissociation rate constant, kd (1/s), using the BIAcore T200 Evaluation Software version 2.0 (Cytiva, Marlborough, MA, USA). The equilibrium dissociation constant was calculated as KD (M) = kd/ka.

## 3. Results

### 3.1. Viscosity Characterization for Two Antibody Variants

**Figure 1:**
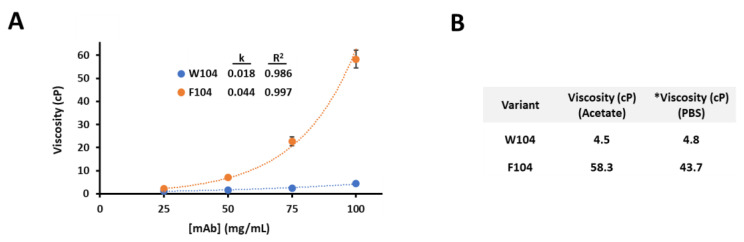
(**A**) Viscosity (dynamic) versus mAb concentration for W104 (blue circles) and F104 (orange circles) IgG1 mAb variants in 20 mM Sodium Acetate pH 5.5 from 25–100 mg/mL. Each viscosity curve was fit to the equation y = Ae^kx^, where the exponential factor (k) and R^2^ of the fit are given for each variant. (**B**) Table of viscosities for the two W104 and F104 mAb variants in both 20 mM Acetate pH 5.5 and PBS pH 7.4 at 100 mg/mL. * Previously reported [6].

Previously, a strongly self-associating mAb was reported where mutagenesis at a single position, HC-CDR3-104, dramatically affected the degree of self-interaction [6]. In Figure 1A, viscosity curves up to 100 mg/mL in a representative low pH and ionic strength formulation buffer (20 mM Sodium Acetate pH 5.5) are plotted for both the HC-W104 and HC-F104 variants. While the W104 variant has low viscosity up to 100 mg/mL (4.2 cP), the F104 variant increases exponentially up to 58.3 cP. At 100 mg/mL in PBS pH 7.4, viscosities were 4.8 and 43.7 cP, respectively, for the HC-W104 and HC-F104 variants (Figure 1B). These measured viscosities in both acetate and PBS buffers are over 2 times higher than what would be considered a typical allowable limit for either downstream processing or injectability [54]. Such strong and robust self-interaction rendered this F104 variant undevelopable, especially for a higher formulation concentration (>100 mg/mL). Since the HC-F104 variant had unacceptably high viscosity in both acetate and PBS buffers, further evaluation of the self-interaction herein focused on PBS formulations for all variants to remain consistent across all biophysical and analytical assays.

### 3.2. Biophysical Characterization and Modeling of Antibody Self-Association Using Individual Antibody Domains for Two Antibody Variants

**Figure 2:**
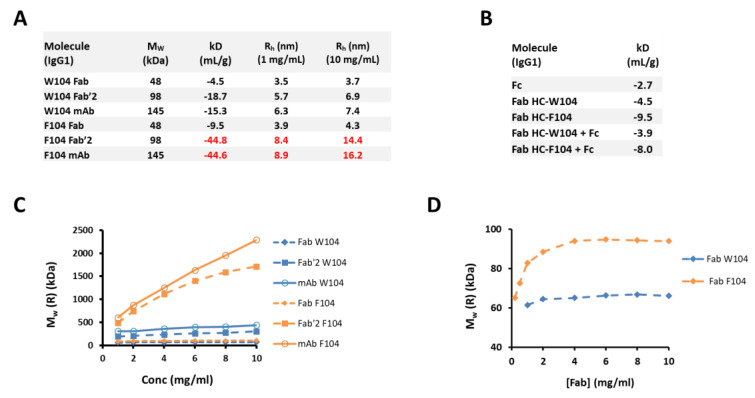
Dynamic Light Scattering (DLS) results for various constructs and HC-104 variants. (**A**) Tabulated molecular weight (M_W_), known from sequence, and interaction parameter (kD) and hydrodynamic radius (R_h_) obtained by DLS for both W104 and F104 variants in the Fab, Fab’2, and mAb forms. Highly negative kDs and large R_h_ values are in red font. (**B**) kD tabulated for Fc and Fab constructs for W104 and F104, and Fab/Fc mixtures (1:1 molar ratio). (**C**) Calculated M_w_ values derived from measured diffusional coefficients plotted against concentration for Fab, Fab’2, and mAb constructs for F104 (orange symbols) and W104 (blue symbols). Fab, Fab’2, and mAb are designated as diamonds, squares, and open circles, respectively. (**D**) Calculated M_w_ values derived from measured diffusional coefficients for Fab forms only of W104 (blue diamonds) and F104 (orange squares).

To better understand the nature of this self-association, individual domains consisting of Fab, Fab’2, and mAb, along with the conserved IgG1 Fc, were prepared for both F104 and W104 variants. In PBS pH 7.4, preparations ranging from 1–10 mg/mL of each construct and variant were prepared and evaluated by DLS. In Figure 2A, construct M_W_ (based on sequence), kD obtained by DLS, and hydrodynamic radius (R_h_) at both 1 and 10 mg/mL, are tabulated. As expected, the F104 mAb self-associated efficiently and a highly negative kD of −44.6 mL/g was obtained. By comparison, the HC-W104 mAb variant, while structurally similar to the HC-F104 mAb, has a significantly less negative kD of −15.3 mL/g. The F104 Fab’2 had a similar highly negative kD of −44.8 mL/g versus its mAb counterpart. This similarity alone is strong evidence that the Fab domains are self-interacting independent of the Fc domain, and further, in Figure 2B, a 1:1 mixture of IgG1 F104 Fab (kD = −9.5 mL/g) and IgG1-Fc (kD = −2.7 mL/g) does not increase self-interaction (or decrease kD), but rather an intermediate kD value results (−8.0 mL/g), supporting the idea that the Fab and Fc domains are not directly interacting.

Interestingly, the F104 variant, which has a high propensity to self-interact, has a much more negative kD in the Fab’2 and mAb forms than the Fab alone (−9.5 mL/g). Likewise, the highly negative kDs for Fab’2 and Mab constructs yield huge, reversible complexes by DLS, resulting in R_h_ values of 14.4 and 16.2 nm at 10 mg/mL in PBS, respectively. Since the sizes of these complexes are not the result of aggregation driven by unfolding (aggregation measured by SE-UPLC is not significant, See Table S1) and are concentration dependent, these are reversible complexes formed through the participation of fully folded, native antibody or antibody domains consistent of typical self-interaction phenomena [28]. The sizes of the complexes formed at 10 mg/mL, a relatively low formulation concentration, for the F104 Fab’2 and mAb constructs are so large that molecular weights of 1714 and 2290 kDa (Figure 2C) are calculated from the R_h_ values (assuming a standard spherical model), respectively. This corresponds to an approximate average size of the 17 non-covalently associated units for the Fab’2 construct and 16 non-covalently associated units for the mAb construct at 10 mg/mL. However, in the case of the F104 Fab construct, only a slightly negative kD of −9.5 mL/g is measured, and R_h_ modestly increases from 3.9 nm to only 4.3 nm in the 1–10 mg/mL concentration range. At 6 mg/mL, a concentration where size appears to have already plateaued, a complex with a M_w_ of 95 kDa is calculated, which approximates a Fab dimer. This highlighted in Figure 2D.

**Figure 3:**
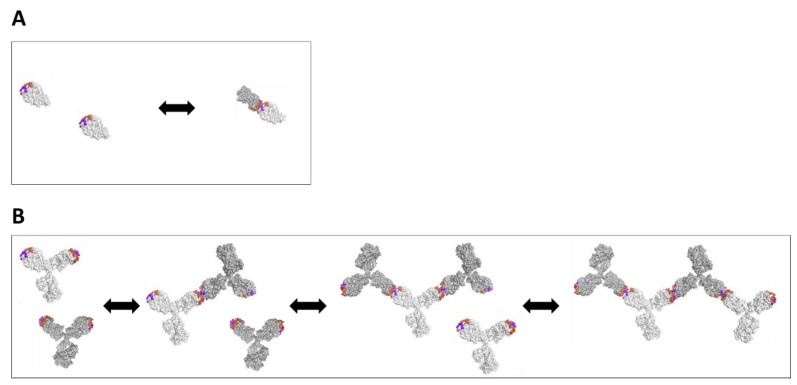
Model depicting (**A**) face-to-face interaction of the Fab forms yielding a dimer/monomer equilibrium and (**B**) bivalent mAb form yielding oligomerization and larger structures that alter bulk solution rheology (up to a tetramer shown).

While the Fab and Fab’2 surely interact in the same manner independent of the Fc domain, they result in entirely different complexes at the same concentrations. From this result, we hypothesized that the interaction was a face-to-face blocking interaction involving the variable domains or HC/LC CDR network, which in the case of the monovalent Fab, would block any subsequent interactions. In the bivalent Fab’2 or mAb forms, however, one face-to-face interaction involving two molecules would still leave two available Fab domains for self-interaction and further self-oligomerization, and this holds true as the polymerization continues to arise in larger complexes in a concentration-dependent manner. In Figure 3, we highlight these proposed models that describe the self-interaction of the Fab domains that simply result in a dimer (Figure 3A), as well as the Fab’2 or mAb constructs that results in a growing oligomeric complex (Figure 3B). In the latter case, such a bivalent arrangement has the potential to dramatically affect rheological properties, and indeed in the case for the F104 mAb variant, incredibly large complexes are formed at merely 10 mg/mL in PBS pH 7.4 and high viscosities are achieved at or below 100 mg/mL (Figure 1).

### 3.3. Evaluation of Self-Binding by BIAcore and ITC

**Figure 4:**
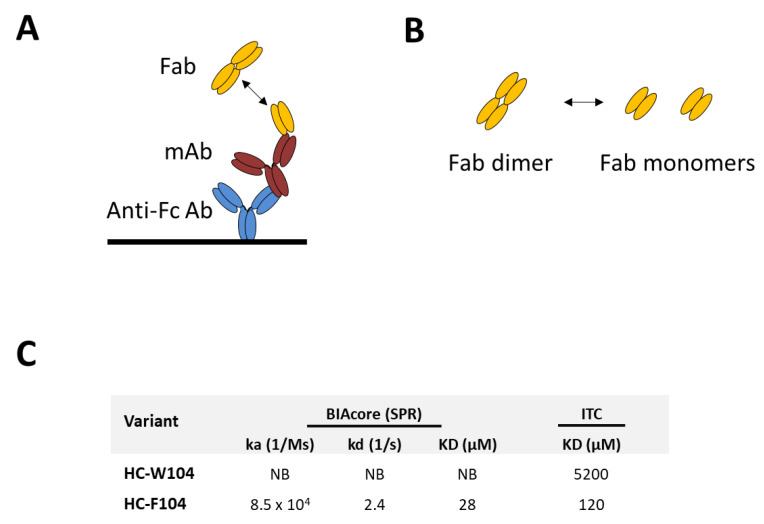
Schematics diagramming (**A**) BIAcore Surface Plasmon Resonance (SPR) format where the mAb serves as the ligand and the Fab of interest, shown here as an equilibrium of monomer and dimer forms, as the analyte. (**B**) Isothermal Calorimetry (ITC) format (in solution) where the Fab of interest is shown as an equilibrium of Fab dimer and monomer. In the experiment, the Fab is dimerized and is fast diluted to initially favor the monomer. (**C**) Measured affinities or KDs (nM) are tabulated for both approaches, including kd and ka for SPR.

To evaluate the nature of this strong self-interaction from a binding perspective, antibody self-binding measurements were obtained using both a BIAcore (SPR) monovalent affinity assay and solution ITC (See Figure 4). In Figure 4A, the BIAcore experiment is diagrammed. Here, both the mAb and Fab were utilized; the mAb served as the ligand conjugated to a chip-bound anti-Fc antibody while the Fab was flowed as the analyte. Therefore, monovalent interactions between the Fab analyte and the mAb ligand could be measured. This was performed for both the HC-W104 and the strongly self-associating HC-F104 variant. By BIAcore SPR, no affinity was obtainable for the weak HC-W104 self-interaction, while a KD of 28 μM was measured for the HC-F104 variant (Figure 4C). This result is consistent with obtained DLS data for the F104 Fab, which was already partially self-associated at 1 mg/mL or ~20 μM. Using ITC, diagrammed in Figure 4B, W104 and F104 Fab preparations in PBS were fast diluted and the change in heat transfer in kcal/mol was measured over time. For the W104 Fab, a very weak KD of 5200 μM (5.2 mM) was obtained; for the F104 Fab, a KD of 120 μM was obtained. Similar to the monovalent self-affinity obtained by BIAcore SPR, the self-binding measured by solution ITC was in the low μM range. In the strongly self-interacting F104 system, a low μM monovalent self-affinity yields a highly negative diffusion interaction parameter obtained by DLS (−44.6 mL/g) and high viscosity at 100 mg/mL (43.7 cP).

### 3.4. Homology Modeling by Patch Analysis and Self-Docking

We performed structure-based modeling studies to explore potentially predictive and corrective tools, as well as in an effort to understand the dramatic difference in self-association observed between the F104 and W104 antibodies. Homology models of the F104 and W104 Fabs were built and subsequently used for computing hydrophobic and charged patches. Attraction between complementary patches, such as two hydrophobic patches or two oppositely charged ionic patches, can drive self-interaction and aggregation [29].

**Figure 5:**
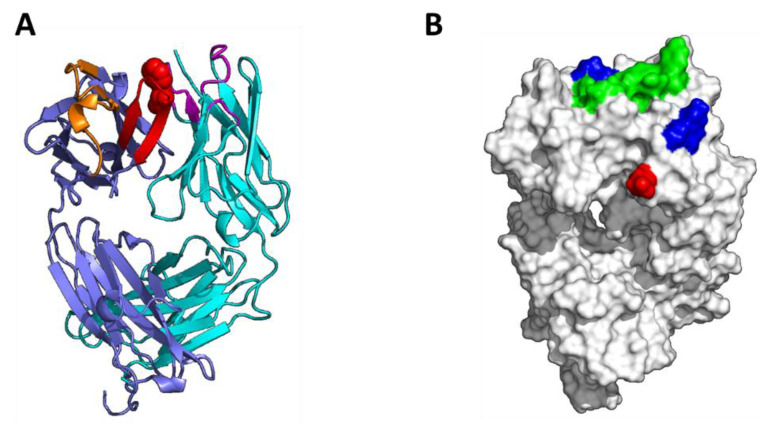

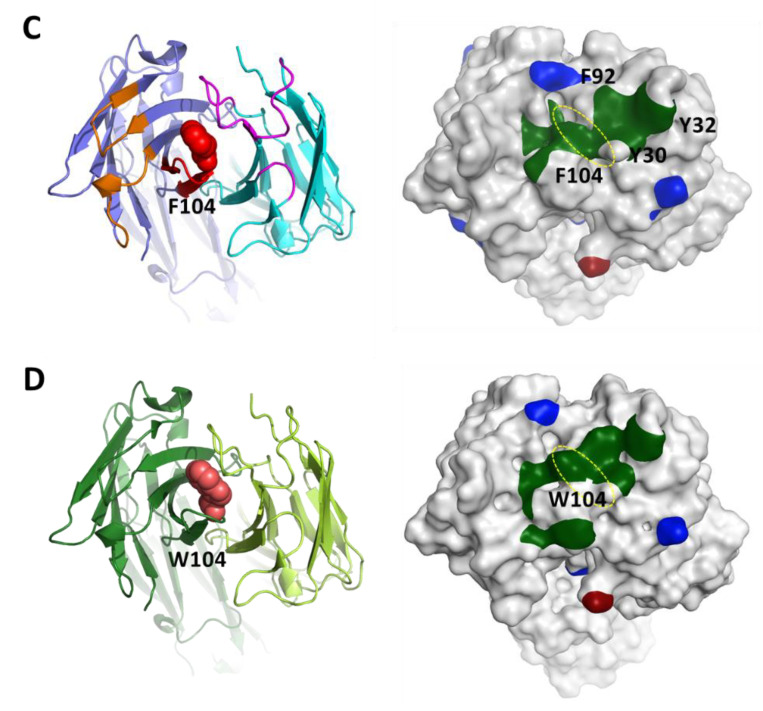
Fab homology models. (**A**) Model of HC:F104 Fab with VL in teal, VH in blue, VL CDR1-3 in purple, VH CDRs 1–2 in orange, VH CDR3 in red, and F104 in red spacefill. (**B**) Surface patch analysis of HC-F104 Fab homology model with hydrophobic patches in green, positive patches in blue, and negative patches in red. (**C**,**D**) Top view of CDR region of HC-F104 Fab and HC-W104 Fab homology models, respectively, rendered in Pymol. Left, ribbon depiction with F104 or W104 highlighted in red spacefill. Right, patch analysis rendered in MOE. F104 and W104 are circled with a dashed yellow ellipse.

Analysis of the F104 Fab model identified a large hydrophobic patch with a surface area of 250 Å^2^ that includes HC-F104 itself and eight additional residues in the LC and HC CDR regions (see Figure 5). This patch includes residues from multiple CDR loops in both the HC and LC, including HC-CDR3, LC-CDR1, and LC-CDR3. In evaluating this prominent hydrophobic patch, the top residues in order of contribution were HC-F104, LC-F92, LC-Y30, and LC-Y32.

Analysis of the W104 Fab homology model identified two hydrophobic patches in the CDR region with areas of 230 Å^2^ and 60 Å^2^. While the total hydrophobic patch size in the W104 antibody CDR region is larger than that of the F104 antibody, at 290 Å^2^ versus 250 Å^2^, the larger side chain of W104 and the presence of a polar NH moiety on the W104 side chain results in a disruption of the hydrophobic patch, which alone may contribute to a reduction in self-interaction in addition to changes in self-complementarity. Further, the larger W104 patch qualitatively differs from the single F104 patch, appearing more branched and discontinuous. In evaluating these patches in the W104 model, HC-W104, LC-F92, LC-Y30, and LC-Y32 are also found to be the top contributors to the overall hydrophobicity.

**Figure 6:**
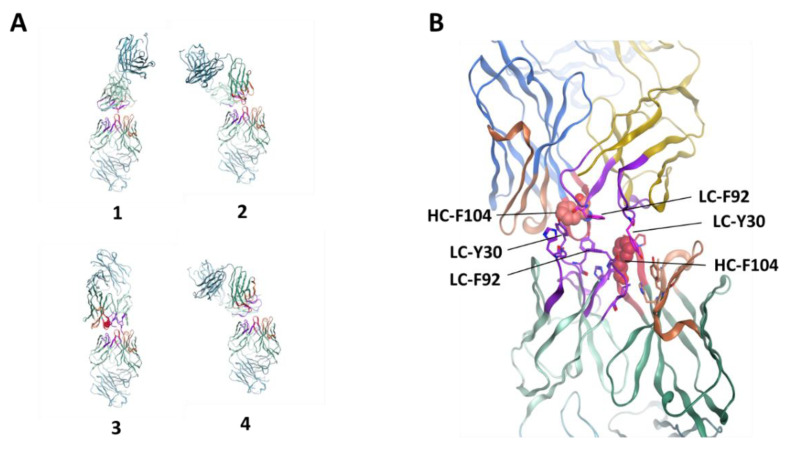
Fab protein-protein docking results. (**A**) Four poses seen in the top epitope cluster. (**B**) Details of model #3, a model for self-interaction supported by experimental data.

To generate hypotheses on specific molecular interactions contributing to the observed self-interaction, we performed protein–protein docking using two modeled F104 Fabs. Docking identified four plausible models for the interaction, which are shown in Figure 6. All models present a face-to-face interaction involving the HC- and LC-CDR apparatus. We prioritized model #3 because it has the largest interaction interface between the two Fabs. The model involves a nearly symmetric interface, with LC-Y30 and LC-F92 of one Fab forming stacking and hydrophobic interactions with HC-F104 of the opposing Fab. We note that docking with the W104 Fab model resulted in similar results, and so docking by itself is unable to rank order the two mutants.

### 3.5. Mutagenesis of Residues Revealed by Homology Model Patch Analysis and Self-Docking

**Figure 7:**
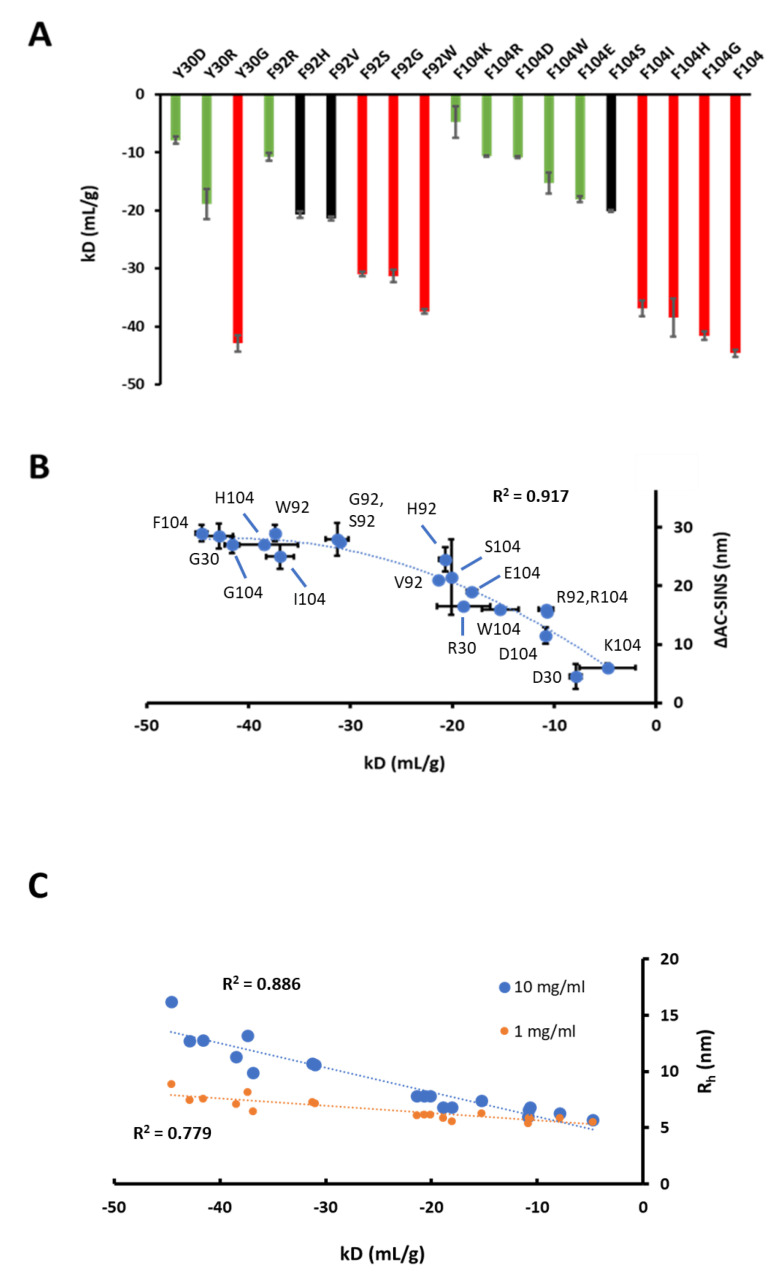
(**A**) kD (mL/g) obtained by DLS (1–10 mg/mL in PBS) shown as bar plots for all variants engineered at HC-104, LC-Y30, and LC-F92. Obtained kD values less than −30 mL/g are shaded red bars, and those higher than −20 mL/g are shaded green. Values between −20 and −30 mL/g are shaded black. Error bars are shown. (**B**) kD values obtained for all 19 interface mutants are plotted against corresponding ∆AC-SINS values (blue circles with error bars for each method and the R^2^ of the parabolic fit). Each data point is labeled by variant. (**C**) kD values obtained for all 19 interface mutants are plotted against corresponding R_h_ values (nm) obtained at both 1 mg/mL (orange circles) and 10 mg/mL (blue circles) along with R^2^ of the linear fits.

Position HC-104 clearly has a large impact on self-interaction and viscosity (Figure 1 and Figure 2). In addition to this position, the preferred homology model revealed two additional residues involved in a prominent hydrophobic patch, LC-Y30 and LC-F92. A fourth residue, LC-Y32, was also identified but not was further evaluated in this study. To verify that these residues do indeed affect self-association in solution, various single mutants at positions LC-Y30 and LC-F92 were engineered on the strongly self-associating HC-F104 variant followed by expression and purification. Additional single point HC-104 mutants were also evaluated. Using purified variants, preparations of 1–10 mg/mL in PBS were evaluated by DLS. In Figure 7A, kD values obtained by DLS for all variants are plotted. Clearly, mutations at all sites had a significant impact on self-association, and some seemingly disrupted self-interaction to baseline kD values, similar to weakly associating W104 Fabs (−4.5 mL/g) and mAbs (−15.3 mL/g), as summarized in Figure 2A. These variants include LC-Y30D (−7.9 mL/g), LC-F92R (−10.8 mL/g), and HC-W104K (−4.8 mL/g), all of whose charged-based mutations were expected to disrupt the self-binding interface and significantly reduce the kD magnitude from the original value of −44.6 mL/g. Other mutations at position HC-104 to Arg and Asp also dramatically reduced the magnitude of kD values. These variants with low and acceptable kD’s, plotted in Figure 7A, are shaded green. Interestingly, mutagenesis to Gly at all three sites resulted in largely negative kD values and only are slightly reduced in magnitude compared to the HC-F104 variant. Other mutations had a moderately reducing effect on self-association (e.g., HC-F104S and LC-F92H, shaded in black). Clearly, the self-interaction of this antibody was dramatically attenuated by single point mutations engineered across all three sites in both the HC- and LC-CDRs.

All variants were also evaluated by AC-SINS, a nanoparticle-based screening assay that utilizes only microgram quantities of material per sample. In PBS, AC-SINS delta wavelength red shift values relative to buffer-only controls were obtained and plotted against the corresponding kD values obtained for each variant. In Figure 7B, obtained kD values and AC-SINS strongly correlate, and linearly correlate, for variants of low or moderate self-association properties (at ranges of approximately −5 to −20 mL/g for kD or 5–20 nm for AC-SINS). For variants that strongly self-associate, AC-SINS correctly predicts this outcome for all variants but is less sensitive to differences in this group, as kD obtained by DLS seemingly is, therefore, flattening the correlation at highly negative kD values (−30 mL/g or less). Therefore, a parabolic correlation is observed between kD and AC-SINS across a very large range of kD values (−4.8 to −44.6 mL/g) obtained in this study for 19 total variants. In Figure 7C, these obtained kD values all linearly correlate with obtained R_h_ values at both 1 and 10 mg/mL, suggesting that self-association can be screened or evaluated by DLS using a single-point concentration measurement as opposed to generating several datapoints to obtain kD.

Although the mutagenesis of key residues was primarily done to evaluate their true effect on self-interaction, from a developability perspective, engineering as a means to correct for undesirable self-association should also maintain desirable target binding affinity at a minimum. In Table 1, the monovalent affinities acquired by SPR against the target antigen are reported. The variant with high and unacceptable self-association, HC-F104, had a sub-nanomolar affinity of 0.35 nM. While several charge-based mutations were made primarily to break self-interaction, such as LC-Y30R, LC-F92R, and HC-F104D, other mutations were more conservative, such as LC-F92W. In this panel, where over 20 single-point mutations were evaluated, the vast majority either yielded non-binding variants or those with markedly reduced SPR monovalent binding to the target antigen. Changes or reductions in KD (nM) were generally driven by reductions in the dissociation rate (kd). The only mutation where binding is strictly maintained or improved was HC-F104W, where SPR binding is slightly improved from 0.35 to 0.16 nM. This mutation also dramatically reduced self-association (kD was reduced from −44.6 to −15.3 mL/g). Other mutations bound with decreased target binding, such as LC-Y30R (12.3-fold decrease), LC-Y30H (15.6-fold decrease), and LC-F92W (15.6-fold decrease), yet also remained in the low nM range. However, if such mutations also dramatically reduced self-association and low nM range target binding affinity was desirable or acceptable, such variants would be reasonable developability candidates. In the case of LC-Y30R, self-association was dramatically reduced to an acceptable level (kD = −18.9 mL/g) and low nM affinity (4.36 nM) was achieved. A similar variant, LC-Y30H, also achieved low nM affinity target binding, but with higher self-association (R_h_ of 11.2 nm at 4 mg/mL versus 6.5 nm at 4 mg/mL for LC-Y30R), meaning it would not likely be as developable, but insufficient material was purified to obtain a full kD plot. A full list of average R_h_ values obtained by DLS for all variants is available in Table S2.

## 4. Discussion

Here, strong self-interaction is observed for HC-F104 mAb constructs, resulting in significantly high viscosities at 100 mg/mL in both low formulation pH and physiological pH conditions and highly negative self-interaction parameters by DLS (see Figure 1 and Figure 2). While formulation dependence is important to self-association propensity, and why, in the case of the HC-F104 mAb, viscosity remains high and even increases at a lower pH and ionic strength (versus PBS pH 7.4) is of interest, this was not explored further in this study. Since high viscosity was found to persist in these formulations that differ by ~2 pH units and have different ionic strengths, we hypothesized that the self-interaction was mainly hydrophobically driven. Additionally, since self-association was robust in PBS, a buffer that is physiologically and process-relevant, is likely to better conformationally stabilize the molecule, and accommodates all experimental approaches herein; all further experimental work was performed in PBS buffer to be consistent across variants and analytical methods.

In Figure 2, it is shown by DLS measurements that individual F104 Fab and Fab’2 domains self-interact independent of the Fc domain. For the W104 variants, self-interaction is dramatically reduced, although a weaker self-interaction persists, and consistent with this observation, low viscosity up to 100 mg/mL across different buffer conditions is observed (Figure 1). For the F104 domain variants, self-association is dramatically enhanced in the bivalent mAb/Fab’2 forms versus the monovalent Fab form, since each self-interaction event leads to additional available CDR faces to propagate oligomerization (see Figure 3). On the other hand, F104 Fab self-interaction leads to dimerization, blocking all additional interactions. Since the HC-CDR3-104 position greatly modulated this interaction and subsequent oligomerization was blocked in the Fab form, a face-to-face interaction involving the CDR regions was modeled (see Figure 3). Interestingly, similar observations were previously made, where rheological differences between monovalent Fab and bivalent Fab’2/mAb have been observed [28,55], although herein we show specifically that the F104 Fab self-dimerizes and further oligomerization is blocked. In terms of the development of bispecifics or multi-specifics, this implies that screening self-association in the bivalent, monospecific forms prior to formatting may not translate at all to rheological effects in the multi-specific form, which is typically monovalent for each targeting arm. This also clearly showcases the challenges of developing even higher order monospecific, multivalent antibody therapeutics (such as tetravalent), which, if appreciably self-interact, will be even more susceptible to high viscosity and negative rheological outcomes directly arising from inherent multi-valency.

By evaluating binding affinities by both BIAcore SPR and ITC, we see that our self-interacting F104 antibody has low μM affinity (28 μM by a monovalent SPR assay and 120 μM by ITC), which appears to be largely driven by high dissociation rates (kd) in comparison to antigen binding (See Figure 4). Self-association of W104 variants are significantly weaker, and either low or non-binding is measured by ITC and SPR, respectively. For the F104 mAb, a low μM self-affinity is significant when considering that concentrations during process and formulation conditions may reach into the mM range, meaning that all recombinant antibodies will be self-associated and oligomerized into large, heterogenous structures. Therefore, we see exponentially high viscosity (43.7–58.3 cP) up to 100 mg/mL (or ~700 μM), precluding higher process and formulation concentrations and further rendering the molecule high risk and undevelopable. This also clearly explains the low viscosities observed for the W104 mAb (4.5–4.8 cP, Figure 1), which self-binds in the mM range, according to ITC. Previously, binding by ITC and BIAcore has been evaluated for a self-interacting antibody system [56], but no affinities were obtained. Only recently, self-binding affinities for strong self-interacting antibodies or antibody binding fragments have been measured using AUC and ITC methods [21]. Similar to the results obtained by Schrag and coworkers for Fab variants, our obtained binding affinities for Fab and/or mAb interactions were in the low μM range. However, in our study, we further correlate these low μM affinities to high viscosities under typical formulation conditions, which clearly render the molecules high risk or undevelopable. Interestingly, our obtained range of binding affinities of 28–120 μM for the F104 Fabs are comparable to other protein–protein interactions that are biologically relevant, such as signaling proteins involved in macromolecular complexation or homodimerization [57,58].

Patch analysis of a preferred homology model of the F104 Fab shows a prominent hydrophobic patch (250 Å^2^, Figure 5), which is consistent with surface areas of other antibodies known to self-interact [16,21]. Within this hydrophobic patch, three hydrophobic, aromatic residues are present: HC-F104, LC-Y30 and LC-F92. We clearly showed that mutations to all 3 sites dramatically impacted self-association characteristics. Consistent with a predominantly hydrophobic self-binding interface, charge-based mutations drastically suppressed self-interaction. Therefore, patch analysis of the F104 Fab correctly predicted the hydrophobic patch primarily responsible for self-interaction and was helpful in identifying the residues most responsible for it. However, this same exercise reaches a similar conclusion when performed for the W104 Fab, which does not appreciably self-interact in solution. In other words, clearly the patch(es) defined by homology modeling can inform which residues to engineer to correct for potential self-association, but by itself cannot predict actual self-association. In addition to likely requiring a defined patch potentially amenable to self-binding, antibody self-complementarity is a clear requirement for self-binding that arises in negative rheological outcomes. This is well underscored by the strong propensity of F104 mAbs or Fab’2s to self-interact, whereas a highly conservative Phe to Trp mutation exhibits a completely different outcome. Perhaps the more discontinuous nature of the patches generated for W104 offers clues to the difference in self-association outcomes. More likely responsible for the great differences between the W104 and F104 variants is the presence of sensitive, tunable self-complementarity analogous to antibody–antigen interactions, where even conservative single point mutations can affect target binding affinities orders of magnitude [59]. In our self-interacting system, we see the large differences between F104 and W104 by both SPR and ITC binding, as well as by DLS and AC-SINS, highlighting how highly specific self-interaction is affected by a simple F104 to W104 transition. Further, both these variants have sub-nM binding against the same biological target (Table 1), therefore they are likely to exist in similar conformations. Interestingly, the mutagenesis of a similar Trp in the HC-CDR3 of another antibody has been shown to dramatically affect self-association [32], highlighting the importance of aromatic CDR residues in self-association. Other aromatic and hydrophobic CDR residues have been reported to be involved in antibody self-association as well [21,55], underscoring that self-association is often driven by forces beyond electrostatics.

The docking of F104 Fabs (see Methods), followed by epitope clustering, resulted in the identification of one dominant epitope, represented by four binding modes, all involving a face-to-face CDR interaction involving the F104 of both Fabs (Figure 6A). Previously, docking has been studied in relation to antibody–antigen or ligand binding [60,61]. In our study, one particular dock model reveals an interaction involving the same patch shown to affect face-to-face self-interaction in solution (Figure 6B). We already mentioned that self-interaction is likely to proceed by a face-to-face arrangement; docking allows us to build atomic models of possible specific interactions consistent with this.

Fab–Fab self-interactions have been characterized in the context of evaluating crystal contacts of pre-existing structures of Fabs and Fab–antigen complexes, where interactions were observed between the Fab CDR region and the Fab Framework (FW) [16,62]. In other reports, Fab–Fc interactions that are isotype dependent have been observed [56]. Additionally, Schrag and coworkers solved a crystal structure of a self-association prone Fab dimer and the dimer interface was mediated by symmetric CDR interactions composed of predominantly aromatic contacts [21]. Our face-to-face self-interaction is also driven by multiple CDR aromatic residues and is likely to be a common configuration and mechanism, since the CDR apparatus has evolved and is designed for binding. Moreover, the top ranked docking structure with the highest buried surface area is a highly symmetrical face-to-face complex involving the CDR apparatus. Consistent with our biochemical and modeling results, Fab self-association reported by Schrag and coworkers were attributed to the binding symmetry and surface flexibility of the CDR apparatus [21]. Interestingly, mutagenesis to glycine at all three CDR sites only slightly lowered self-association, indicating that, while the self-binding interface is highly specific to certain interfacial residues, it may be compensated through increased local main chain flexibility, enabled by the presence of glycine residues. Additionally, the inherent binding proclivity, along with inherent flexibility of the CDRs, means that the self-binding of the CDR apparatus to itself is inevitable and one of the likely possibilities for self-interacting recombinant antibodies.

From a developability perspective, there is significant interest in understanding how to better predict and correct self-association at a sequence or in silico level without impacting other key attributes such as binding and stability. Here, simply using a homology model, three sites were revealed in a prominent hydrophobic patch and all were shown to significantly impact self-interaction. To be viable correction variants, at a minimum target antigen, binding affinity or activity must be acceptably maintained. In Figure 7, only one variant out of the 22 total variants that were mutated at three separate CDR sites, HC-W104F, maintains both sub-nM target antigen affinity and significantly lowers self-interaction to an acceptable range. Another variant, LC-Y30R, also significantly lowered self-association to an acceptable level and maintained low nM target affinity. This exercise demonstrates the difficulties of correcting problematic self-association, particularly when the same CDR apparatus that has evolved for binding is also involved in strong self-interaction. Often, high affinity, or significantly improving it through affinity maturation, increases the likelihood of further encountering negative developability attributes [21,63]. Supporting this notion, in the case of the HC-F104 variant, we have both sub-nM target binding and unacceptable self-association propensity. Because antibody self-interaction will almost certainly involve the functional CDR apparatus, possibly on both molecules, the probability of success for each variant to maintain tight target binding and reduce self-association is low (<10% in our study). Therefore, we have learned that the selection and evaluation of more variants at various positions will increase the likelihood of achieving the desired results. Overall, it is shown here that both homology patch analysis and self-docking can inform potential self-associating regions with low μM affinity and residues amenable to corrective engineering despite its difficulty, but also may not predict actual self-association because of the apparent requirement for unique self-interaction specificity or complementarity between molecules.

## 5. Conclusions

Herein, reversible antibody self-association is evaluated biochemically using individual domains, through binding affinity measurements, by in silico homology modeling and docking approaches, and by site-directed mutagenesis. Interestingly, the self-interaction of mAb variants are shown to likely proceed through a blocking face-to-face interaction involving the HC and LC CDRs that is potently magnified by bivalency. This self-binding, leading to unacceptable viscosity at 100 mg/mL in different formulations, also has low μM self-affinity and is highly tunable by and sensitive to single-point mutations to three different HC/LC CDR residues informed by homology model patch analysis and self-docking. The evaluation of these self-association mutants demonstrate that corrective engineering is difficult yet feasible and warrants several mutations at multiple positions to increase likelihood of success. Additionally, in silico approaches, while useful and informative in identifying potential self-interacting regions, are likely not able to address the specificity and complementarity of self-binding that is evident in a system that exhibits significant self-interaction propensity and sensitivity to even conservative mutations at multiple sites. This study enhances our understanding of antibody self-association and potential means of addressing and mitigating it from a developability perspective.

## Figures and Tables

**Table 1:** BIAcore SPR and DLS results summary table for all variants. Tabulated are SPR KD (nM) values, the ratio of the measured KD and the HC-F104 reference KD (KD/KD_ref_), and kD (mL/g) values obtained by DLS. The reference F104 is highlighted gray. All LC-Y30 and LC-F92 variants are site-directed mutants of the F104 variant. The single F104W variant with comparable BIAcore SPR KD to the reference is highlighted green. Variants with 10–20× differences in binding relative to the variant are highlighted yellow, and those with greater than 20-fold difference in SPR binding or non-binding (NB) are highlighted red. kD (mL/g) measured by DLS is color coded in terms of degree of self-association (greater than −20 mL/g, green; −20 to −29 mL/g, yellow; −30 mL/g or less, red). “NA” denotes no measurement.

Variant	KD (nM)	KD/KD_ref_	kD (mL/g)
F104	0.35	1	−44.6
F104W	0.16	0.44	−15.3
F104I	108	305	−36.9
F104H	1372	3879	−38.5
F104D	NB	NB	−10.9
F104K	NB	NB	−4.8
F104E	NB	NB	−18.1
F104S	NB	NB	−20.1
F104G	NB	NB	−41.6
F104R	NB	NB	−10.7
Y30R	4.36	12.3	−18.9
Y30H	5.5	15.6	NA
Y30N	45.5	129	NA
Y30D	60.9	172	−7.9
Y30Q	82.6	234	NA
Y30G	170	481	−42.9
F92W	5.51	15.6	−37.4
F92H	35.1	99.2	−20.7
F92V	38.7	109	−21.4
F92R	50.2	142	−10.8
F92S	62.7	177	−31
F92G	142	402	−31.3

## Data Availability

Not applicable.

## Supplementary Materials

The following are available online at https://www.mdpi.com/2073-4468/10/1/8/s1, Table S1: SE-UPLC retention times (RT) and % purity tabulated for each variant, Table S2: Tabulated diffusional coefficients (Do), hydrodynamic radii (Rh) measured over the range of 1–10 mg/mL, and ∆AC-SINS values (in nm) for all variants evaluated. “NA” denotes not measured or available.
