# Supplementary material for: Characterization and Modeling of Reversible Antibody Self-Association Provide Insights into Behavior, Prediction, and Correction
Source: Antibodies (Basel). 2021 Feb 15;10(1):8. doi: 10.3390/antib10010008 (PMC7931086; doi:10.3390/antib10010008)
Supplement: Supplementary file 1 [file antibodies-10-00008-s001.pdf]

Variant	SE-UPLC RT (min)	% Purity
F104	2.34	98.1
F104W	2.48	92.7
F104I	2.47	97.7
F104H	2.46	98.3
F104D	2.39	98.5
F104K	2.46	98.1
F104E	2.43	89.2
F104S	2.45	89.7
F104G	2.40	91.1
F104R	2.43	91.5
Y30R	2.37	93.1
Y30H	2.29	93.8
Y30N	2.33	86.3
Y30D	2.37	94.9
Y30Q	2.31	93.0
Y30G	2.26	93.6
F92W	2.45	90.2
F92H	2.39	90.5
F92V	2.39	90.3
F92R	2.40	92.2
F92S	2.37	89.7
F92G	2.35	90.0

Table S1. SE-UPLC retention times (RT) and % purity tabulated for each variant.

		R_h values (nm)						
**Variant**	**D_o (cm^2/s)**	**1 mg/mL**	**2 mg/mL**	**4 mg/mL**	**6 mg/mL**	**8 mg/mL**	**10 mg/mL**	**ΔAC-SINS**
**F104**	2.51E-07	8.9	12.1	13.0	14.0	15.3	16.2	29
**F104W**	3.87E-07	6.3	6.8	6.9	7.0	7.3	7.4	16
**F104I**	3.78E-07	6.5	7.2	7.9	8.6	9.5	9.9	25
**F104H**	3.35E-07	7.1	8.3	9.2	9.9	10.7	11.3	27
**F104D**	4.60E-07	5.4	5.5	5.6	5.8	5.9	6.0	12
**F104K**	4.56E-07	5.5	5.5	5.5	5.6	5.6	5.7	6
**F104E**	4.39E-07	5.6	5.9	6.1	6.3	6.6	6.8	19
**F104S**	3.96E-07	6.2	6.6	7.0	7.0	7.3	7.8	22
**F104G**	3.09E-07	7.6	9.2	10.1	11.3	11.9	12.8	27
**F104R**	4.17E-07	5.9	6.1	6.2	6.3	6.4	6.8	16
**Y30R**	4.20E-07	5.9	6.2	6.5	6.6	6.8	6.8	17
**Y30H**	2.43E-07	10.8	11.2	12.7	NA	NA	NA	30
**Y30N**	3.32E-07	7.8	8.0	8.9	NA	NA	NA	30
**Y30D**	4.20E-07	5.9	6.0	6.0	6.3	6.3	6.3	5
**Y30Q**	3.48E-07	7.5	8.1	9.3	NA	NA	NA	28
**Y30G**	3.08E-07	7.5	9.3	10.5	11.9	12.2	12.7	29
**F92W**	2.87E-07	8.2	10.3	10.2	11.4	12.3	13.2	29
**F92H**	3.96E-07	6.2	6.7	6.9	7.1	7.5	7.8	25
**F92V**	4.00E-07	6.1	6.6	6.9	7.1	7.5	7.8	21
**F92R**	4.22E-07	5.9	6.0	6.1	6.3	6.4	6.6	16
**F92S**	3.28E-07	7.2	8.4	9.1	9.3	10.1	10.6	28
**F92G**	3.24E-07	7.3	8.5	9.2	9.5	10.1	10.7	28

Table S2. Tabulated diffusional coefficients (D_o), hydrodynamic radii (R_h) measured over the range of 1-10 mg/mL, and ΔAC-SINS values (in nm) for all variants evaluated. "NA" denotes not measured or available.
